# Aging and cancer epigenetics: Where do the paths fork?

**DOI:** 10.1111/acel.13709

**Published:** 2022-09-14

**Authors:** Raúl Fernández Pérez, Juan Ramón Tejedor, Agustín Fernández Fernández, Mario Fernández Fraga

**Affiliations:** ^1^ Cancer Epigenetics and Nanomedicine Laboratory Nanomaterials and Nanotechnology Research Center (CINN‐CSIC) El Entrego Spain; ^2^ Health Research Institute of Asturias (ISPA‐FINBA) Institute of Oncology of Asturias (IUOPA) and Department of Organisms and Systems Biology (BOS) University of Oviedo Oviedo Spain; ^3^ Rare Diseases CIBER (CIBERER) Carlos III Health Institute (ISCIII) Madrid Spain

**Keywords:** aging, cancer, DNA methylation, epigenetic clock, stem cells

## Abstract

Aging and cancer are clearly associated processes, at both the epidemiological and molecular level. Epigenetic mechanisms are good candidates to explain the molecular links between the two phenomena, but recent reports have also revealed considerable differences, particularly regarding the loss of DNA methylation in the two processes. The large‐scale generation and availability of genome‐wide epigenetic data now permits systematic studies to be undertaken which may help clarify the similarities and differences between aging and cancer epigenetic alterations. In addition, the development of epigenetic clocks provides a new dimension in which to investigate diseases at the molecular level. Here, we examine current and future questions about the roles of DNA methylation mechanisms as causal factors in the processes of aging and cancer so that we may better understand if and how aging‐associated epigenetic alterations lead to tumorigenesis. It seems certain that comprehending the molecular mechanisms underlying epigenetic clocks, especially with regard to somatic stem cell aging, combined with applying single‐cell epigenetic‐age profiling technologies to aging and cancer cohorts, and the integration of existing and upcoming epigenetic evidence within the genetic damage models of aging will prove to be crucial to improving understanding of these two interrelated phenomena.

## Graphical abstract

Aging and cancer are interrelated processes which share common epigenetic alterations. Nonetheless, there are differences in some of the DNA methylation changes which occur in both phenomena. The recent development of epigenetic clocks will help dissect the common and specific epigenetic characteristics of aging and cancer, although the mechanisms underlying epigenetic clocks are yet to be clarified, particularly in relation to somatic stem cell epigenetic aging.

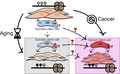

**Abbreviations**
5mC: 5‐methylcytosine
CpG: cytosine‐guanine dinucleotide
DNAm: DNA methylation
ESC: embryonic stem cell
HSC: hematopoietic stem cell
MSC: mesenchymal stem cell

## 1 INTRODUCTION

Aging, a quasi‐universal biological phenomenon, is inextricably linked to the development of cancer (de Magalhães, 2013). Understanding the mechanisms underlying the causative role that this gradual, time‐dependent accumulation of molecular damage plays in disease etiology is crucial to designing interventions seeking to improve our healthspan—that is, the lifespan free of disease. In this scenario, the field of aging research—and aging epigenetics in particular—is experiencing an explosive revolution owing to the development and democratization of high‐throughput technologies and computational methods (Hasin et al., 2017), which has led to the large‐scale profiling of sizable cohorts using genome‐wide tools. Against this background, we can now re‐examine the available evidence regarding the molecular parallelisms and differences between aging and cancer in order to shed light on the relationship between the two processes.

Classically, parallel epigenetic alterations have been described for aging and cancer, mainly concerning the local DNA hypermethylation of CpG islands which is typically marked by bivalent chromatin domains in embryonic stem cells (ESCs) (Day et al., 2013; Easwaran et al., 2012; Fernández et al., 2015; Heyn et al., 2012; Ohm et al., 2007; Rakyan et al., 2010; Schlesinger et al., 2007; Teschendorff et al., 2010; Widschwendter et al., 2007) and the global loss of DNA methylation (DNAm), particularly at repetitive sequencies (Bollati et al., 2009; Ehrlich, 2009; Fuke et al., 2004; Li et al., 2014; Wilson et al., 1987).

Nonetheless, these similitudes have recently been challenged, particularly in terms of DNA hypomethylation, by studies using next‐generation sequencing technologies to measure 5mC at the genome‐wide level (Unnikrishnan et al., 2018). This has been especially the case with research involving mouse models of aging, which have failed to report global DNA hypomethylation in this process, across multiple tissues (Cole et al., 2017; Hadad et al., 2019; Hahn et al., 2017; Hernando‐Herraez et al., 2019; Masser et al., 2017; Sun et al., 2014). These observations have prompted systematic studies to be undertaken that seek to accurately characterize the similitudes and differences between aging‐ and cancer‐associated epigenetic alterations, often revealing clear differences between aging, cancer, and senescence (Pérez et al., 2018; Xie et al., 2018), some of which have been detectable across human and mouse (Pérez et al., 2021). There is therefore a need to clarify, within the epigenetic context, the functional relationships between the two processes.

Aside from the canonical hyper‐ and hypomethylation signatures of aging and cancer, the recent development of epigenetic clocks (Horvath & Raj, 2018) has provided us with a new dimension in which to explore aging‐associated epigenetic dysregulation and its link with carcinogenesis. In this work, we explore the current knowledge regarding the DNAm alterations classically described in aging and cancer and consider epigenetic clocks as novel, albeit different, tools which may help clarify the complex relationship between the two processes, paying particular attention to the phenomenon of somatic stem cell epigenetic aging.

## 2 THE ROLE OF CLASSIC AGING‐ASSOCIATED DNA METHYLATION ALTERATIONS IN CANCER

There appear to exist a number of functional connections between aging and cancer that involve epigenetic mechanisms. Especially with regard to DNA hypermethylation, it seems that the genomic loci which experience aging‐associated gains in DNAm in healthy tissue are in turn aberrantly hypermethylated in cancer (Lin & Wagner, 2015; Luebeck et al., 2019), which would seem to indicate that tumors present a “hyperaged” phenotype that could be associated with cancer risk and survival (Klutstein et al., 2017; Lin & Wagner, 2015). These epigenetic alterations can be linked to mitotic activity and, indeed, epigenetic mitotic clocks have been developed by tracking DNAm gains at specific loci (Yang et al., 2016). In this same line, more mechanistic studies have shown how spontaneous epigenetic lesions accumulated over time can facilitate oncogenic transformation, for example, in mouse colorectal organoids through the repression of key genes (Tao et al., 2019). Of course, time‐associated alterations may also reflect the accumulation of lifestyle‐related aggressions: for instance, long‐term exposure to cigarette smoke condensate has been shown to produce epigenetic alterations leading to tumorigenesis in human lung cells (Belinsky et al., 2002; Vaz et al., 2017). With respect to the loss of DNAm, the commonalities between aging and cancer again appear to be related to cell division, and DNA hypomethylation has been observed at late‐replicating, lamina‐associated domains in both aging and cancer, with cancer once again manifesting stronger alterations (Dmitrijeva et al., 2018; Zhou et al., 2018). However, the functional impact of the gradual loss of methylation at these gene‐poor, heterochromatin associated loci remains to be fully clarified: while aging‐associated hypermethylation gives rise to alterations which may indeed facilitate the oncogenic process, the canonical global hypomethylation often seen in tumors could actually be a consequence, and not a cause, of the dramatic cellular expansion occurring after malignant transformation.

Thus, it would seem that an important functional epigenetic link between aging and cancer relates to DNAm alterations, which partly reflect the mitotic history of the tissues involved—an “accelerated” history in the case of tumors—and, particularly regarding DNA hypermethylation, these changes may functionally influence the oncogenic process by dysregulating the expression of cancer‐related genes. Nonetheless, it is possible that there exist other aging‐associated epigenetic alterations which have an impact on cancer development but are otherwise not present, or non‐detectable, in tumoral tissues: (1) subtle aging changes could be masked, or reverted, by global epigenetic reconfiguration in tumors (Pérez et al., 2018); (2) aging‐related alterations in the tumor microenvironment may have oncogenic roles (Marks et al., 2016); (3) epigenetic changes in the immune system brought about by immunosenescence could also modify the risk of, and response to, cancer in more indirect ways (Pawelec, 2017).

In addition, other aging‐associated phenomenon may contribute to concealing the putative functional characteristics of aging‐related epigenetic alterations, including the existence of a stochastic component inherent to age‐associated DNAm alterations (Seale et al., 2022) and the occurrence of age‐associated changes in cellular composition (Campagna et al., 2021). Nonetheless, the phenotypic and epigenetic variability brought about by aging via mechanisms such as tissue disruption may in itself causally contribute to the oncogenic process, as has been recently proposed (Capp & Thomas, 2021). To tackle these concerns, systematic meta‐analysis studies focusing on multi‐tissue common alterations in aging and cancer (Chen et al., 2016; Pérez et al., 2018) and also the use of single‐cell epigenetic profiling technologies (Cheung et al., 2018; Hernando‐Herraez et al., 2019) are clearly needed.

## 3 DNA METHYLATION CLOCKS AS NEW TOOLS IN THE STUDY OF DISEASE

The past decade has been witness to a revolution in the field of aging epigenetics: the development of epigenetic clocks. Epigenetic clocks are mathematical algorithms which can, with great precision, predict the chronological age of subjects by combining measured DNAm levels at multiple CpG sites of their genome (Horvath & Raj, 2018). There is now clear evidence of the association between alterations in the predictions made by epigenetic clocks and lifestyle factors, disease—including cancer—or outright mortality (Fransquet et al., 2019; Levine et al., 2018; Lu et al., 2019; Marioni et al., 2015; Oblak et al., 2021), indicating that these algorithms capture a combination of chronological and biological age, the latter being a measure of the “healthiness” of an individual in terms of their risk of developing age‐associated adverse outcomes (Jylhävä et al., 2017). For instance, supercentenarian or long‐lived subjects, who display reduced incidence, or delayed onset, of diseases (Andersen et al., 2012) also manifest younger epigenetic ages (Armstrong et al., 2017; Horvath et al., 2015). Moreover, there is incipient evidence of epigenetic clock rejuvenation through pharmacological‐ or lifestyle‐based clinical interventions in human (Fahy et al., 2019;Gensous et al., 2020; Noreen et al., 2020; Fiorito et al., 2021; Fitzgerald et al., 2021). Thus, current data support the importance of epigenetic clocks as biomarkers of aging, although studies focusing on the direct modification of clock components will be necessary to demonstrate whether these sets of CpGs can directly modulate the aging process. That said, because these clocks seem to so accurately capture information related to internal or environmental aggression which may be associated with disease, their close examination may help clarify the putative causal relationships between aging and cancer.

### 3.1 Functional meaning of the epigenetic clocks and somatic stem cell aging

What—if any—is the functional meaning of epigenetic clocks? Recent reports have indicated that the CpG sites which make up different clocks often share core functional characteristics in terms of their association with genomic elements or with gene expression levels (Jonkman et al., 2022; Liu et al., 2020), and *in vitro* models have been built that partially link cellular passage clocks with organismal aging (Minteer et al., 2022), supporting the notion that these algorithms are not merely abstract constructs but rather reflect underlying biological processes. Indeed, the intrinsic characteristics of DNAm clocks in relation to their differing “ticking rates” during lifespan, which are accelerated during development and subsequently slow down during aging, (Horvath & Raj, 2018) suggest that they may be particularly linked to an underlying biological system. Stem cells display a youthful—pre‐natal in fact—epigenetic phenotype (Horvath, 2013) and there are experimental observations indicating that the rejuvenation of the epigenetic clock occurs during embryonic development (Kerepesi et al., 2021) and can be achieved through the transient expression of reprogramming factors in human and mouse models (Chondronasiou et al., 2022; Gill et al., 2022; Sarkar et al., 2020). Thus, the ticking rate of the epigenetic clocks could in fact reflect those developmental and tissue‐homeostasis maintenance processes driven by stem cells—and involving alterations in cell composition—that occur throughout life, processes, which can in turn be modified by internal or external factors during aging (Horvath & Raj, 2018; Raj & Horvath, 2020). A recent review summarized three hypotheses regarding the biological processes which could be responsible for the ticking of the clock CpGs whereby the gradual accumulation of DNAm alterations could reflect (Seale et al., 2022): (1) the life‐long tissue turnover via asymmetric stem cell division; (2) the decay of circadian mechanisms during aging, which could be linked to metabolic dysregulation; or (3) the genomic re‐localization of epigenetic enzymes to DNA damage sites, which appear and accumulate with aging. Nevertheless, these systemic views of epigenetic clocks need to be reconciled with recent evidence demonstrating that epigenetic aging can be measured in individual cells (Trapp et al., 2021). This new clock, termed “scAge”, was actually built from bulk‐tissue derived aging CpGs, and thus, fits well with models of aging‐associated increases in tissue heterogeneity (Rudolph, 2021), but the development of higher‐resolution DNAm profiling in single cells will allow for the construction of epigenetic predictors directly from these data. After all, DNAm is a discrete/binary molecular mark, and it remains to be explored whether there is a mechanistic basis to how, or in what order, CpG sites may be altered with age in individual cells.

**FIGURE 1:**
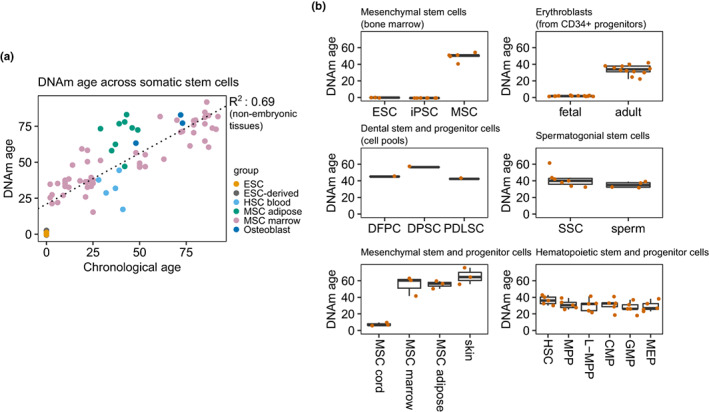
Somatic stem cells and progenitors display epigenetic aging. (a) Scatterplot depicting the correlation between chronological and epigenetic age (Horvath clock) across various types of somatic stem cells and progenitors, embryonic stem cells, and derived embryonic tissues. The R‐squared coefficient and line of fit are given for the correlation involving non‐embryonic tissues (HSC: hematopoietic stem cell, from peripheral blood; MSC: mesenchymal stem cell, from adipose or bone marrow tissue; ESC: embryonic stem cell). (b) Boxplots showing the epigenetic age estimations of different types of somatic stem cells and progenitors for which measurements of chronological age were unavailable (ESC: embryonic stem cell; iPSC: induced pluripotent stem cell; MSC: mesenchymal stem cell; DFPC: dental follicle progenitor cell; DPSC: dental pulp stem cell; PDLSC: periodontal ligament stem cell; SSC: spermatogonial stem cell; HSC: hematopoietic stem cell; MPP: multipotent progenitor; L‐MPP: late multipotent progenitor; CMP: common myeloid progenitor; GMP: granulocyte‐macrophage progenitor; MEP: megakaryocyte‐erythroid progenitor).

In addition, the relationship between epigenetic aging and “stemness”, or plasticity, must be more clearly defined. Adult stem cells, which reside in tissue‐specific niches and are responsible for the maintenance of tissue homeostasis (Cheung & Rando, 2013), show evident signs of aging‐associated decline (Oh et al., 2014), including epigenetic alterations (Bork et al., 2010; Fernández et al., 2015). A recent report that studied hematopoietic stem cell (HSC) transplantation has shown that transplanted HSCs give rise to reconstituted blood which manifests a DNAm age similar to that of the HSC donor (Søraas et al., 2019), suggesting that the initial stem cells carry a specific age signature. Thus, the question of whether somatic stem cells display epigenetic clock aging still needs to be clarified. To tackle this issue, we compiled DNAm data from various independent studies profiling different types of somatic stem cells and progenitors (see Methods for full list of datasets) and determined their epigenetic ages using the Horvath clock (Horvath, 2013). Our results clearly show that somatic stem cells display DNAm clock aging across a wide range of tissues (Figure 1 a‐b), as opposed to embryonic stem cells and fetal tissues. In an epigenetic clock model, it makes sense that aging somatic stem cells accumulate a DNAm age signature which is then propagated to tissues during homeostasis and regeneration. Studies that dissect somatic stem cells at the single‐cell level in particular are needed to clarify this issue, and there is indeed an example of muscle stem cells showing low epigenetic ages in mouse (Hernando‐Herraez et al., 2019), but more data is clearly required.

### 3.2 Alterations in epigenetic clocks and the epigenetic age of tumors

With regards to the alterations—acceleration or deceleration—observed in the clocks, various associations have been investigated, particularly the aforementioned links with environmental factors and disease (Fransquet et al., 2019; Levine et al., 2018; Lu et al., 2019; Marioni et al., 2015; Oblak et al., 2021), to ascertain whether the DNAm alterations which occur during these processes could drive the changes observed in the clocks. For instance, a recent study investigating DNAm age acceleration in the non‐tumoral breast tissue of breast cancer patients demonstrated that the observed alteration in the epigenetic clock could be explained by a subset of the clock CpGs that suffer from the well‐known cancer‐associated hypermethylation of Polycomb associated loci (Rozenblit et al., 2022). Indeed, clocks have been constructed which partly reflect the DNAm alterations induced by cancer‐related lifestyle factors such as smoking (Lu et al., 2019). There are also genetic factors, some of which also regulate the biology of the aging process, which are associated with the ticking rate of epigenetic clocks (Lu et al., 2018; McCartney et al., 2021). However, a recent report using Mendelian randomization methods to explore the possible causal role of epigenetic clocks in cancer development has demonstrated that there were few causal associations between the ticking rates of these clocks and increased cancer risk (Morales Berstein et al., 2022). Thus, at this stage there is little evidence to suggest that epigenetic clocks are primary drivers of aging or age‐related disease as opposed to being biomarkers that reflect the influence of genetic or environmental factors, or other underlying processes (Drew, 2022). Nonetheless, it may also be that some form of cancer‐specific clock dysregulation with functional meaning occurs directly during the process of oncogenic transformation, meaning that the tracking of age acceleration in healthy tissues, as done in most studies, would not yield valuable insight.

**FIGURE 2:**
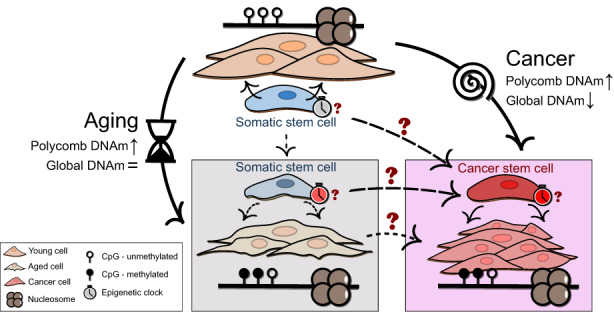
Unresolved questions regarding the links between aging and cancer DNAm alterations. There are several loose ends with regards to how DNAm alterations may constitute mechanistic links between aging and cancer. The classic aging‐DNAm alterations include Polycomb‐associated hypermethylation, which also occurs in cancer, but the similarities with respect to hypomethylation are less clear as tumors display much clearer global hypomethylation, which could be related to their drastic expansion, while aging‐associated loss of DNAm is linked to different chromatin signatures. On the other hand, epigenetic clocks seem to be accelerated in cancer in spite of the increased cellular plasticity and activity of cancer cells. It remains to be explained if and how somatic stem cells display epigenetic clock aging, and how this phenomenon may influence cancer development, in particular with regards to cancer stem cells.

In addition to the aforementioned findings, we should consider the following observations (Figure 2): (1) the classic aging‐associated DNAm alterations described in studies are very different from clock‐associated DNAm changes with aging: clock‐DNAm changes with age are actually very subtle (Horvath, 2013) and the CpG sites involved could in fact be considered “stable enough” to be able to track time‐dependent alterations in spite of internal or external assaults occurring through lifespan, while aging‐DNAm changes are much stronger and specific in terms of their biological associations (Day et al., 2013; Fernández et al., 2015; Pérez et al., 2018), and, moreover, there are even aging‐DNAm signatures which accelerate with age (Sziráki et al., 2018); (2) as already mentioned, aging DNAm signatures—especially DNA hypermethylation—are typically augmented in cancer (Lin & Wagner, 2015; Luebeck et al., 2019) to the extent that, in this sense, tumors may appear to have an aged phenotype.

On the other hand, we know little regarding the specific reconfiguration of the clock DNAm within tumoral cells: they appear to also have an hyperaged phenotype (Horvath, 2013; Pérez et al., 2018) but this could be related to cancer‐associated dramatic hypermethylation events affecting a subset of the clock CpGs. In fact, tumors such as thyroid cancer, for which age is actually a prognosis indicator (Kazaure et al., 2018), show reduced clock‐DNAm dysregulation—i.e. they age more “naturally” (Horvath, 2013; Pérez et al., 2018; Yang et al., 2016). It is possible that the age acceleration observed in tumors may partly be caused by epigenetic clocks capturing the mitotic, proliferative history of tissues, which is evidently amplified in cancer. This issue has been tackled by comparing the behavior of a mitotic clock and the Horvath clock in the context of B‐cell tumors to show that epigenetic age can sometimes be independent of epigenetic proliferative history (Duran‐Ferrer et al., 2020), thus suggesting that there may be mechanisms underlying epigenetic clocks which are not entirely connected with cellular division. Furthermore, if we acknowledge that tumoral cells—and not exclusively cancer stem cells—have an increased quality of stemness, or plasticity, which increases their proliferation and dissemination potential (Batlle & Clevers, 2017), should we expect them to have a higher or lower epigenetic age? There is evidence that some stem‐related traits such as telomerase expression do not lead to the rejuvenation of the epigenetic clock (Lu et al., 2018), while, as mentioned previously, the induction of pluripotent reprogramming factors does (Chondronasiou et al., 2022; Gill et al., 2022; Sarkar et al., 2020).

Altogether, it seems that aging‐DNAm and clock‐DNAm dysregulation present distinct molecular features, and more studies, again particularly those focusing on the single‐cell dissection of epigenetic aging in healthy and tumoral tissues, will be needed to clarify these issues.

## 4 FUTURE DIRECTIONS: GENETIC AND EPIGENETIC AGING

It is becoming increasingly accepted, in what is known as the “somatic mutation” theory of aging (Kennedy et al., 2012), that the unifying mechanism of aging is the time‐dependent accumulation of DNA damage (Schumacher et al., 2021). Indeed, there are recent compelling results suggesting that mutational rates may define lifespan across mammalian species (Cagan et al., 2022), and that this could be partly controlled by differences in the efficiency of DNA repair mechanisms (Gorbunova et al., 2014; Tian et al., 2019). In this same line, recent observations indicate that aging‐associated epigenetic dysregulation—including epigenetic clock acceleration—could be a consequence of DNA damage via the re‐localization of epigenetic modifiers during DNA repair (Hayano et al., 2019; Kane & Sinclair, 2019). This would suggest that features of epigenetic aging may be brought about by the accumulation of aging‐associated DNA damage and, indeed, the stochastic accumulation of genomic alterations is consistent with the epigenetic noise typically observed during the aging process (Tejedor & Fraga, 2017). The recent development of multispecies epigenetic profiling technologies (Arneson et al., 2022) is starting to provide insight into these questions. New studies conducted by the Mammalian Methylation Consortium have provided very interesting results that support the notion that aging mechanisms are evolutionarily conserved: (1) DNA methylation clocks have been successfully built for the prediction of age across mammalian species that may be associated with developmental processes (Lu et al., 2021); (2) epigenetic markers of maximum lifespan have also been developed which are quite different from aging‐associated DNAm alterations (Li et al., 2021), although new analytical approaches can be used to define CpG sites that integrate information from both aging and lifespan, which may be particularly useful as biomarkers of anti‐aging interventions (Haghani et al., 2022).

Results from epigenetic studies often point towards the existence of development‐associated mechanisms underlying the universal features of aging; however, they will have to be reconciled with the existing genetic evidence positing that the accrual of DNA damage is the main driver of this process. Furthermore, with regards to the links between aging mechanisms and the development of cancer, they will have to be examined in the light of recent powerful evidence indicating that, while cancer is also a universal disease across mammals, differences in cancer risk across species are largely independent of longevity—and body mass—and could be related to lifestyle factors such as diet (Vincze et al., 2022). Thus, epigenetic mechanisms may still hold the key to revealing how universal biological processes such as aging or cancer— which may in themselves be reflected through DNAm biomarkers—are modulated by environmental, non‐genetic factors.

## 5 METHODS/DATASETS

To explore the dynamics of DNAm aging in somatic stem cells, we retrieved DNAm values in the form of beta values or intensity measurements from 13 publicly available array‐based datasets: GSE52114 and GSE17448, containing mesenchymal stem cells (MSCs) from bone marrow (Bork et al., 2010; Fernández et al., 2015); E‐MTAB487, containing CD34 + hematopoietic stem cells (HSCs) from peripheral blood (Bocker et al., 2011) (reanalyzed samples of MSCs from GSE17448 were removed); GSE202067, containing MSCs and osteoblasts from bone marrow (Giesche et al., 2022); GSE138311, containing MSCs from adipose tissue (Serena et al., 2020) (only healthy subjects were used); GSE26519, containing MSCs from adipose tissue (Schellenberg et al., 2011; 5‐passage samples were used while redundant 10passage samples from the same individuals were removed); GSE116754, containing embryonic stem cells (ESCs) and derived embryonic tissue (Colunga et al., 2019); GSE34688, containing MSCs from bone marrow, derived induced pluripotent stem cells (iPSCs) and ESCs (Shao et al., 2013); GSE56491, containing erythroblasts derived from CD34 + progenitors from fetal liver and adult bone marrow (Lessard et al., 2015); GSE112933, containing periodontal stem cells (pooled from various patients); GSE72444, containing spermatogonial stem cells and sperm samples (Struijk et al., 2020); GSE41933, containing MSCs from adipose tissue, bone marrow, umbilical cord, and dermal fibroblasts (Reinisch et al., 2015); and GSE63409, containing various hematopoietic progenitors (Jung et al., 2015).

Data were handled with the R statistical software (v4.0.5) and graphs were constructed using the *ggplot2* package (v3.3.3; Wickham, 2016). DNAm ages were estimated using the Horvath clock (Horvath, 2013) via the *ENmix* package (v1.26.10; Xu et al., 2021).

## AUTHORS CONTRIBUTION

Raúl Fernández Pérez, Juan Ramón Tejedor, Agustín Fernández Fernández, and Mario Fernández Fraga conceived, coordinated, and supervised the study. Raúl Fernández Pérez designed all aspects of the research, collected the data, performed analyses, and wrote the manuscript. All authors revised, read, and approved the final manuscript.

## FUNDING INFORMATION

The Spanish Association Against Cancer (grant number PROYE18061FERN to MFF), the Asturias Government (PCTI) co‐funding 2018‐2022/FEDER (grant number IDI/2018/146 to MFF), Fundación General CSIC (grant number 0348_CIE_6_E to MFF), the Health Institute Carlos III (Plan Nacional de I + D + I) co‐funding FEDER (grant number PI21/01067 to MFF and AFF) and the Spanish Ministry of Science and Innovation (grant number SGL2021‐03‐039/40 to M.F.F.) co‐funding NextGenerationEU. JRT is supported by a Juan de la Cierva fellowship from the Spanish Ministry of Science and Innovation (grant number IJC2018‐36825‐I). RFP is supported by the Severo Ochoa program (grant number BP17‐114). We also acknowledge support from IUOPA‐ISPA‐FINBA (the IUOPA is supported by the Obra Social Cajastur‐Liberbank, Spain) and Consorcio Centro de Investigación Biomédica en Red (CIBERER‐ISCIII).

## CONFLICT OF INTEREST

None declared.

## Data Availability

Data sharing is not applicable to this article as no new data were created or analyzed in this study.
